# Quantifying simultaneous innovations in evolutionary medicine

**DOI:** 10.1007/s12064-020-00333-3

**Published:** 2020-11-25

**Authors:** Deryc T. Painter, Frank van der Wouden, Manfred D. Laubichler, Hyejin Youn

**Affiliations:** 1 School of Complex Adaptive System, Arizona State University, Tempe, AZ 85281 USA; 2 Department of Geography, University of Hong Kong, Pok Fu Lam, Hong Kong; 3 Santa Fe Institute, Santa Fe, NM 87501 USA; 4 Kellogg School of Management, Northwestern University, Evanston, IL 60208 USA; 5 Northwestern Institute on Complex Systems, Evanston, IL 60208 USA; 6 London Mathematical Lab, London, WC2N 6DF UK

**Keywords:** Simultaneous innovation, Independence, Novelty, Keyword extraction, Evolutionary medicine

## Abstract

To what extent do simultaneous innovations occur and are independently from each other? In this paper we use a novel persistent keyword framework to systematically identify innovations in a large corpus containing academic papers in evolutionary medicine between 2007 and 2011. We examine whether innovative papers occurring simultaneously are independent from each other by evaluating the citation and co-authorship information gathered from the corpus metadata. We find that 19 out of 22 simultaneous innovative papers do, in fact, occur independently from each other. In particular, co-authors of simultaneous innovative papers are no more geographically concentrated than the co-authors of similar non-innovative papers in the field. Our result suggests producing innovative work draws from a collective knowledge pool, rather than from knowledge circulating in distinct localized collaboration networks. Therefore, new ideas can appear at multiple locations and with geographically dispersed co-authorship networks. Our findings support the perspective that simultaneous innovations are the outcome of collective behavior.

## Introduction

Innovations provide a wide range of benefits to society and are seen as the key drivers of progress. All throughout modern history, innovations are often contributed to the merits and relentless hard work of individuals, thus receiving credit for the work (Cyranoski et al. 2011; Fiske 2011; Larson et al. 2014; Turk-Bicakci et al. 2014). However, identifying pioneering work and assigning the appropriate credits to the corresponding scholars has not been straightforward in history. Famously, Thomas Kuhn, before publishing his work on scientific paradigms (Kuhn 1962), worked on the issue of simultaneous discovery using the formulation of the principle of energy conservation as an example (Kuhn 1959). One of the most famous examples is the anecdote of Alexander Graham Bell who filed his patent on telephone just before Elisha Gray (Hounshell 1975; Evenson 2015) is now being contested by the revelation of Antonio Meucci’s “speaking telegraph” (Catania 2002; Campanella 2007). Another one in biology is probably with Charles Darwin and Alfred Russell Wallace, who simultaneously formulated the concept of natural selection that laid the ground work for our modern ideas of evolution (Beddall 1968; McKinney 1972; Kutschera 2003; Armstrong 2019).

These examples seem to contradict the believes that innovations are created by a handful of knowledgeable geniuses, tinkering away in isolation (Rahnasto 2003; Varian et al. 2004; Kultti et al. 2006). If innovations were made by individuals themselves, why do we observe so many cases of simultaneous innovations? It is highly unlikely that two geniuses simultaneously produce the same innovation if the idea is such a breakthrough. Moreover, it does not align with the special status these individual innovators are assigned as individuals with unique and exclusive knowledge sets and skills.

Instead, following Francis Galton (Galton 1874), the occurrence of simultaneous innovation suggests that novelty is the product of the collective environment, rather than an attribute of individual genius. According to Galton, innovative discoveries occur naturally when enough resources and scholars are engaged in the problem. The efforts of these scholars push the knowledge frontier closer until innovations ultimately solve the problem. Like water filling up a bathtub, once the water exceeds the edge of the bath it floods at numerous parts simultaneously. Thus, this perspective argues that innovations may very well occur simultaneously not because individual knowledge pools are unique as one might expect from rugged individualism, but because scholars draw from an increasing collective knowledge pool (Merton 1973).

An important premise of this is that scholars can access this pool of knowledge independently. A collective knowledge pool does not exclude the access of scholars but, instead, is publicly accessible. This collective pool is represented, for instance, by the ever-growing library of published academic research papers and, in the digital age, the accessibility of these by everyone and from everywhere in the world. There is no need to be geographically close to the original authors of the knowledge or belong to their social network to access the knowledge. The occurrences of independent simultaneous innovations suggest the presence of a collective knowledge pool.

In this study we investigate the existence of a collective knowledge pool in the field of evolutionary medicine. We examine to what extent simultaneous innovations occur and whether these are independent from each other. However, establishing whether two simultaneous innovations are produced independently is difficult because scholars can be connected across multiple platforms over which they can influence each other. We therefore chose to test for independence by examining whether scholars of simultaneous innovations are connected through collaboration networks, citation behavior, and geographical proximity. To do so, we use a large corpus containing 6, 456 academic papers in the field of evolutionary medicine between 2007 and 2011. We use the novel, persistent keyword framework, first published in (Painter 2019), to identify papers in this corpus that simultaneously introduce innovations. While this previous work focused on the methodology of assigning keywords and testing the validity of the framework, we leverage that work to measure and identify simultaneous, independent innovations. Tests of independence and reliance on a collective knowledge pool are done by evaluating the co-authorship, citation behavior, and geographical dispersion retrieved from the corpus’ metadata.

**Fig. 1:**
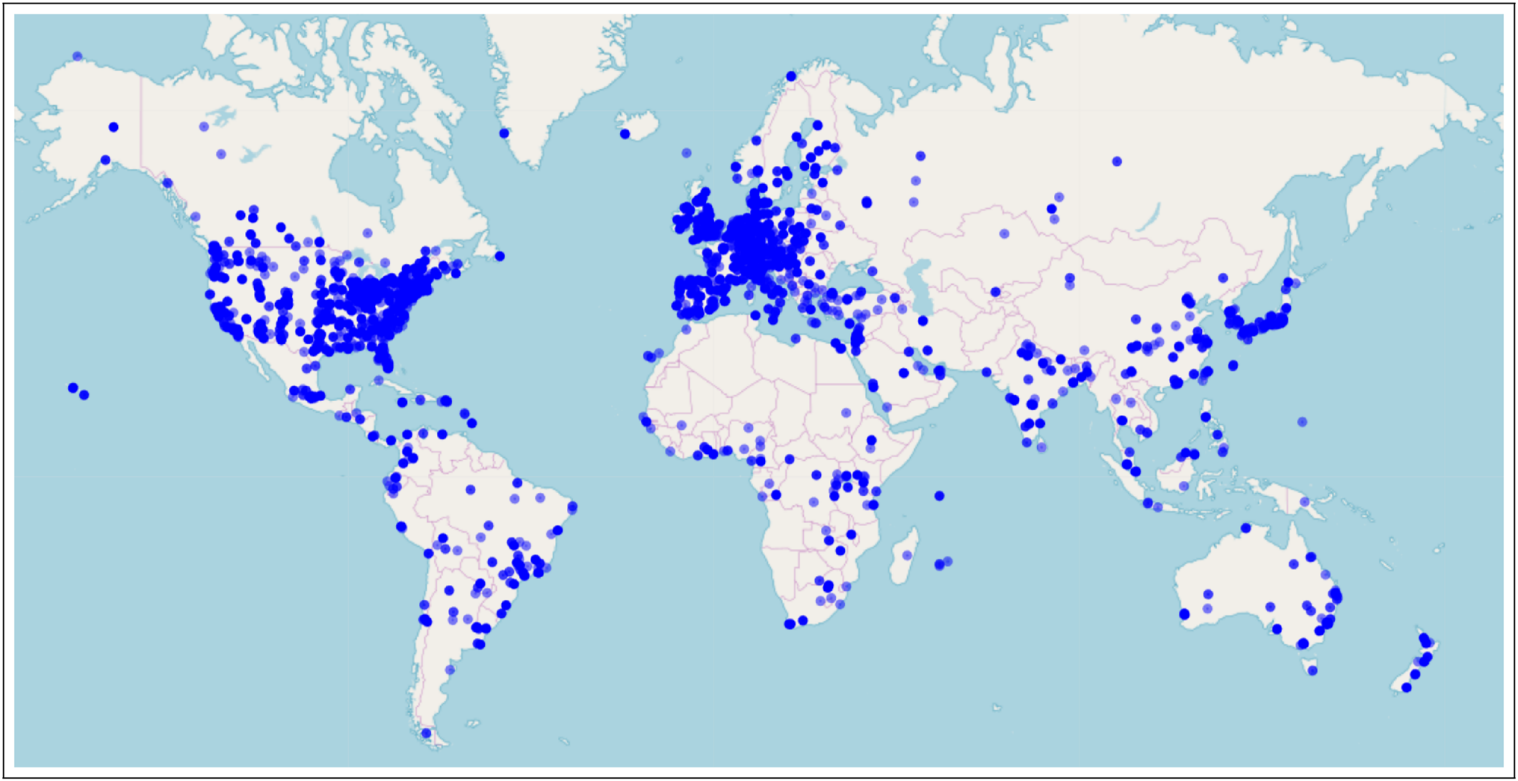
Affiliation location of authors in evolutionary medicine. Created using (Guide and Geocoding 2018)

We focus on the field of evolutionary medicine for three distinct reasons. First, evolutionary medicine is an interdisciplinary synthesis of traditional medical research and evolutionary biology (Williams and Nesse 1991; Nesse and Williams 2012) and is recognized as a distinct, mature, interdisciplinary scientific field (Alcock 2012; Painter et al. 2021). Interdisciplinary is closely linked to innovation and therefore suitable for a study on innovation (Stevenson and Nuottila 2016; Gerullis and Sauer 2017; Pacheco et al. 2017; Delgado and Åm 2018; Gohar et al. 2019). Second, the field has an active global community of researchers and scholars that is registered in a global directory. Using this directory and extending it with data from secondary sources allows us identify and record authors and their connected work in evolutionary biology *written-large*. This variety and platform attract scholars from around the globe, as shown in Fig. 1. Previous work has shown that using the proposed novel, persistent keyword framework has been successful identifying innovations within evolutionary medicine (Painter 2019). Finally, we conclude with an analogy discussion about the conceptual similarities between convergent/parallel biological evolution in ecological niches and dependent/independent knowledge evolution in intellectual niches.

The main result of our study supports the claim of the existence of a collective knowledge pool. Defining innovation on a spectrum founded upon the Schumpeter principles of novelty and persistence (Brozen 1951; Schumpeter 1934, 1939, 1942), we demonstrate how a wide scale methodology for identifying innovation can be leveraged to discover computational foundations within the study of knowledge. These findings suggest that the power to innovate not necessarily purely resides within the individual genius but rather in the collective effort of the scholars in a field to push the boundaries of what is known. We stand on the shoulders of giants. However, we are held aloft by a community.

## Materials and methods

There is active debate about the extent and economic value on the degree to which knowledge recombination is useful for producing innovations (Kaplan and Vakili 2015; Savino et al. 2017; Rhee and Leonardi 2018; Aggarwal and Hwang 2018; Jung 2019; Zhang et al. 2019; Hargadon 2003). However, it is generally accepted among innovation scholars that novelty and innovations are more generally the product of a recombination of previous knowledge in novel ways (Fleming and Sorenson 2004; Murray and O’Mahony 2007; Goldstein et al. 2010; Davis and Eisenhardt 2011; Petruzzelli and Savino 2014; Youn et al. 2015 Kim et al. 2016). In this, individuals or groups of authors act as melting pots by combining their individual knowledge with domain-specific knowledge. (Carey and Spelke 1994; Merton 1973) In this case study, domain-specific knowledge is knowledge relevant to a situation or class of problems. (Dewey 2007) Here, we define the domain-specific pool as knowledge represented by the keywords identified from publications in years prior to the year in question. Explicitly,

1 $$\begin{aligned} K_d=\sum _{i=a}^{b-1}{W_i}, \end{aligned}$$

where *a* is the first year of the corpus, *b* is the year in question, and $W_i$ is the set of keywords extracted from a given year. $K_d$ indicates the collective, domain-specific knowledge available to everyone within that specific domain of research. For example, 2007’s domain knowledge pool consists of all the keywords identified in 2006, 2005, 2004, *etcetera*; 2008’s would include 2007, 2006, and so on. Because we measure innovation at the level of the publication, the knowledge pool for that publication consists of the common collective knowledge pool and the unique knowledge contributed by each collaborator. This can be formally expressed as

2 $$\begin{aligned} K_p=(\sum _{i=1}^n K_i) \cup (K_d+K_c+K_z), \end{aligned}$$

where $K_i$ represents an abstraction of the individual author’s knowledge pool summed over the number of co-authors on the publication to create the authors’ knowledge pool, and $K_c$ is the common knowledge of an average, educated adult. $K_d$ is the fundamental domain knowledge ubiquitous to the specific research field, while $K_z$ represents a kind of knowledge zeitgeist . Together $K_d+K_c+K_z$ sums to the common, collective knowledge pool. The union in 2 indicates the union of the authors’ knowledge pool, $\sum _{i=1}^n K_i$ and the common collective knowledge pool, $K_d+K_c+K_z$ results in $K_p$, the knowledge pool of the publication.

While it might still be possible to identify the individual, original contributions of their knowledge pool—read: previously identified keywords—the end result of the publication is something uniquely different—read: novel, persistent keywords. Or, as Aristotle is oft quoted, “the whole is more than the sum of its parts.”

Common collective knowledge is the information freely available to individuals based on their participation in specialized research. The content of the common collective knowledge pool is contextually and temporally dependent. The common collective knowledge pool of an economist is different than that of a microbiologist which will, in turn, be different for each of them in the future. A common collective knowledge pool consists of common general knowledge, $K_c$, assumed information known by an educated adult, and domain-specific knowledge, $K_d$, specialized knowledge known generally to experts in a field (Walker 1987; Alexander 1992; Hjørland and Albrechtsen 1995). The classification of the knowledge pool, $K_p$, can be formalized as

3 $$\begin{aligned} I(K_p)_x, I(K_p)_y = {\left\{ \begin{array}{ll} \text {Specialized} & \, \text {if} [\text {R}]_x = [\text {R}]_y \,\, \text {and}\,\, [\text {A}]_x \ne [\text {A}]_y \\ \text {Collective} & \,\text {if} [\text {R}]_x \ne [\text {R}]_y \,\,\text {and}\,\, [\text {A}]_x \ne [\text {A}]_y \\ \text {Dependent} & \,\text {otherwise} [\text {A}]_x = [\text {A}]_y \end{array}\right. } \end{aligned}$$

where the knowledge pool of innovations *x* and *y*, $I(K_p)_x$ and $I(K_p)_y$ are classified as “Specialized” knowledge if they do not share any co-authors, [*A*], but share references, [*R*], indicating that both innovations share information from domain-specific knowledge pool, $K_d$. We classify the innovations as arising from a collective knowledge pool, $K_z$, if $I(K_p)_x$ and $I(K_p)_y$ do not share references or co-authors. Finally, if the publications share the same author, they are considered dependent on each other, parts to a single innovation, $I=\sum _{i=1}^{n} I(K_p)_n$.

Science is increasingly becoming a collaborative effort (Greene 2007; Laudel 2001). This is relevant to understand the publication knowledge pool, $K_p$, in that $n=1$ for Equation 2 resulting in a much smaller $K_p$. $K_i$ and $K_d$ are ever evolving summation of previous information. When researchers collaborate, they are contributing to the individual knowledge pools of their collaborators, and, in turn are acquiring knowledge themselves. We can assume some level of knowledge homogenization as information flows freely between authors. However, complete acculturation of individual knowledge pools between co-authors is highly unlikely. In addition, the resulting $K_p$ may or may not contribute to the collective knowledge of a domain, $K_d$, as interdisciplinary research is directional, as shown in (Painter et al. 2021). This means that when an economist and a biologist publish an article in a biology-focused journal, the economist is bringing new knowledge into biology, but the biologist is not bringing knowledge from the biology domain into economics, $E(K_i) \longmapsto B(K_d)$. Therefore, this kind of collaboration is likely to bring novel keywords into the domain of the journal’s audience. Where those keywords to be adopted by other biologists in subsequent years, we would consider the hypothetical publication resulting from said collaboration to be innovative. The future publications producing those same keywords represent the adoption of the novelty thereby supporting our claims of innovation.

Now consider the case where two biologists collaborate on a biology publication and later in the future both individuals come up with the same innovation at the same time. The two innovations are separate, simultaneous innovations as the previous collaboration between the biologists changed their respective individual knowledge pools and contributed to the common collective knowledge of all biologists, thus illustrating the time-sensitive nature of individual and collective knowledge. In addition, the collaboration between the economist and the biologist is more likely to result in innovative keywords being introduced into biology giving a theoretical basis for claims that interdisciplinary research is linked to higher rates of innovation. (Stevenson and Nuottila 2016; Gerullis and Sauer 2017; Pacheco et al. 2017; Delgado and Åm 2018; Gohar et al. 2019)

We use a novel, persistent keyword framework to identify innovations in a large corpus containing academic papers in evolutionary medicine between 2007 and 2011 (Painter 2019). To begin, we identify individuals interested in or working on fields associated with evolutionary medicine. We include individuals who are registered in The International Society for Evolution, Medicine, and Public Health (ISEMPH) global directory. These individuals are scholars, clinicians, students, and community supporters with a self-proclaimed interested in evolutionary medicine (EvMed Network) (Nesse 2018). We also included editors and contributors from two popular evolutionary medicine textbooks. (Trevathan et al. 1999; Gluckman et al. 2009) For each individual we gather their academic publication from Web of Science (Reuters 2012) and transform these into plain text files and then into a comprehensive corpus using the Giles framework. (Damerow et al. 2017) Our full corpus contains 6, 456 full-text publications from 1971 through 2017. The corpus is then hand curated to identify errors, such as wrongly assigning work to individuals. Previous research findings suggest that such ambiguities introduce a negligible amount of error. (Newman 2001; Barabâsi et al. 2002)

**Fig. 2:**
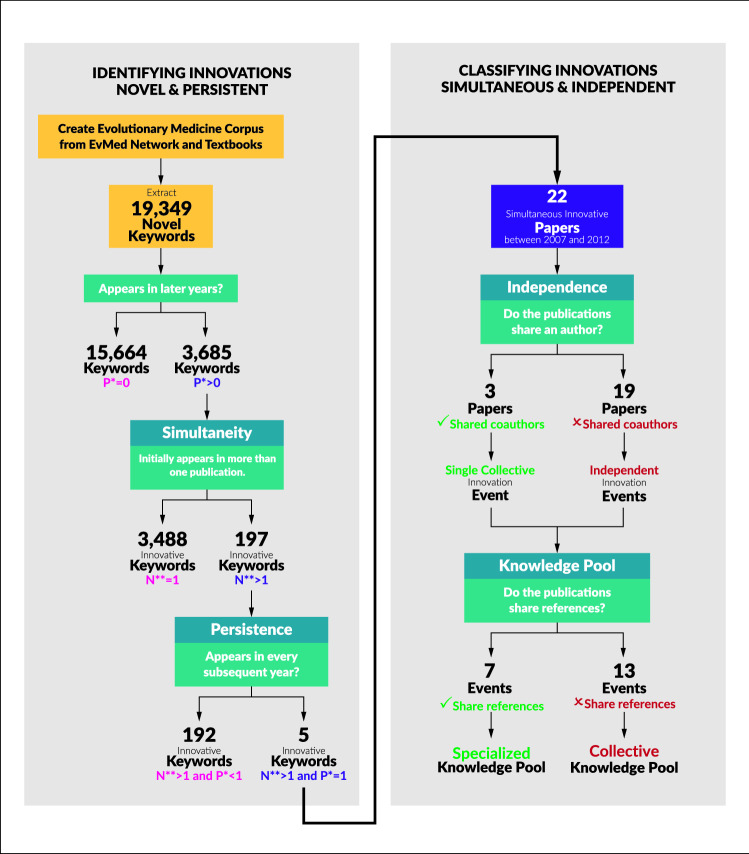
Methodology of identifying innovation, simultaneity and independence. $$P^*$$ is formalized in Equation 5 and $$N^{**}$$ in Equation 4. The first column represents the identification of novel keywords that persist through the years that were introduced by more than one publication. The second column shows how those simultaneous innovations were identified as independent or dependent innovation events based on shared co-authors. Next, their knowledge pools (references) are examined for bibliographic coupling between events

**Table 1:** Classification of simultaneous innovation (SI) papers. Papers that introduce novel keywords that persist every year after are considered simultaneous innovations by virtue of more than one paper introducing a novel persistent keyword in the same year

		Knowledge pool		
**Simultaneous innovation papers**		Local	Collective	Total
	Dependent	3	0	3
	Independent	6	13	19
	Total	7	13	22

SI innovations are considered dependent if they share authors between publications. These publications are later grouped into a single innovation event. A local knowledge pool is defined by bibliographically coupled papers. Independent SI innovations are classified in the collective knowledge pool if they do not share references between the papers *vis-a-vis* the novel, persistent keyword

We include all the academic publications of individuals identified as interested in evolutionary medicine. This means we include publications that might not seem directly affiliated with evolutionary medicine, *per se*. However, we incorporate these publications because they might embody the broader spirit of evolutionary medicine and have the potential to bring-in core tenets of evolution into medicine. These publications are therefore evolutionary medicine in nature if not necessarily by name. The metadata for each publication was downloaded to serve as a reference from the Web of Science. More on this later.

We identify five keywords that were first introduced into the evolutionary medicine multiple times in the same year: metabolites, chromatin, triglyceride, epigenome, and exome. We must call attention to the fact that this is not the first instance of the keyword in the scientific literature. It is merely the first time they appear in the evolutionary medicine corpus. Therein, the introduction and then adoption of said keyword by the evolutionary medicine community as a whole, as evidenced by their reappearance every year afterward, that is tantamount to innovation. The keywords are representative of concepts being incorporated into evolutionary medicine, as measured by their novelty and persistence.

Similar to Painter (2019), we identified instances of innovation by the occurrence of novel keywords that then persist throughout the rest of the corpus. Therefore, *the innovation is the incorporation of new ideas, represented by the novel  keywords, into evolutionary medicine, and not the idea or keyword itself*. An innovative publication contained a keyword that was not previously identified in the corpus and persisted in every following year. Keywords were identified by converting the plain text file of each publication into word frequency lists. Those word frequency lists were then compared to the reference corpus Baker-Brown for General American English (AmE06) (Baker 2012) to identify keywords using WordSmith Tools’ default significance threshold *p*-value of $10^{-6}$. Scott (1999) The Baker-Brown corpus was used as a reference because a reference corpus built specifically for science might in fact exclude words of interest due to their high frequencies in such a corpus. Thus, keywords become those words that appear with a significantly higher frequency than those in the reference corpus.

A publication was characterized as innovative if a new keyword was identified that had not previously been identified in the corpus and said keyword could be identified in every year subsequently. This follows Joseph A. Schumpeter’s and Yale Brozen’s innovation framework involving invention, innovation, and imitation. Brozen (1951) invention implies novelty. Brozen and Schumpeter define innovation as a change in process. Imitation then becomes the adoption by others. The novel, persistent keyword framework incorporates each definition as novelty being the first occurrence of a particular keyword, innovation and imitation are combined into the persistence of said keyword. We feel this is a justifiable means to identify innovation. However, we are aware this methodology is context dependent based on factors such as corpus creation and keyword identification. We created a baseline group of keywords from 1991 until 2006. Therefore, beginning in 2007 we are able to identify novel, persistent keywords by comparing 2007 keywords to the baseline for novelty, and then to each subsequent year to check for persistence. For each subsequent year, the previous year’s keywords are added to the baseline. Again, we must make a note here that the innovations identified using this framework are not the first appearance of this keyword ever. Rather, this is the first appearance of this keyword in evolutionary medicine, and it is therefore influenced by the way the corpus is constructed. The methodology for identifying simultaneous innovation events is illustrated in Fig. 2 and further explained in Table 1.

To identify which publications were involved in simultaneous innovation, we identified novel, persistent keywords that appeared multiple times together in the same year within separate publications. The novelty of a keyword is measured as

4 $$\begin{aligned} N= \# \text { of publications in which a keyword first appears in the same year}, \end{aligned}$$

and a keyword’s persistence is measured as

5 $$\begin{aligned} P=\frac{\# \text {of subsequent years a keyword is found}}{ \#\text { of subsequent years}}. \end{aligned}$$

We are interested in instances of simultaneous innovation; therefore, innovative keywords must possess $N>1$ and $P=1$. This indicates that a keyword first appeared in multiple publications that year and was subsequently found at least once every following year. For the publications containing these keywords, we examined their citations, references, and co-authors to determine if they were indeed independent instances of simultaneous innovations. If the publications contained no co-authors in common and did not cite each other, they were considered separate events. If they contained no references in common, they are considered to have originated using separate common collective knowledge pools. If the publications in question do in fact share references, they are still considered independent and simultaneous, but they are classified as having arisen from the same common knowledge, a product of their environment.

To provide similar data for later statistical tests, we gathered topical content of these innovative publications. Therefore, we identified approximately 100 publications that are similar to the innovative papers based on shared, non-innovative keywords. Each publication has a set of keywords that were extracted from the full text using the workflow mentioned above. Therefore, it was straightforward to compare the keywords between texts and set a threshold for similarity based on how many keywords a publication in question shared with the innovative papers identified from each year in order to create similar sample sizes of the topic in question, approximately 100 similar publications.

For the geographical analysis, we examine the extent to which author(s) of innovative and similar non-innovative papers concentrate in space. If authors of innovative papers are more likely to concentrate in space as compared to authors of non-innovative papers, this might suggests that specific, localized knowledge is required to produce innovations. To examine this we associated each innovative paper to geographic locations based on author(s) affiliation, retrieved from the Web of Science metadata. These affiliations were then geo-coded using Google’s Geo-coding API (Lemke et al. 2015; Xu et al. 2012; Guide and Geocoding 2018). The same is done for 472 non-innovative papers that have similar characteristics in terms of non-innovative keywords and number of co-authors, as compared to the innovative papers. We then find the average geographical distance of the five nearest co-authors for the innovative papers in each year. We use bootstrapping tools to randomly select the same number of non-innovative papers from our pool of similar non-innovative papers in that year and find the average geographical distance between the authors on these papers. We repeat this exercise 50,000 times to construct a distribution of expected average geographical distance between authors in the field of evolutionary medicine. This allows us to make statistical inference on the likelihood for the observed geographical distribution of authors of innovative papers to occur at random.

## Results

**Table 2:** Average citation counts for all publications of a given year, innovative publications of said year, and publications that share non-innovative keywords in the same year

Year	Keyword	Yearly average citations	Average innovation citations	Average similar citations
2007	Metabolites	75	52	112
2008	Chromatin	43	146	78
2009	Triglyceride	31	205	114
2010	Epigenome	34	127	180
2011	Exome	55	221	97

2007 is the only year where the average citation count was lower than both the yearly and similar average. 2010 also shows an average innovative citation count lower than the similar publication citation count

Previous research (Painter 2019) has shown that using a novel, persistent keyword framework to identify and rank innovative publications by the number of novel, persistent keywords introduced into the evolutionary medicine corpus were significantly more probable to accumulate more citations per year than the average evolutionary medicine publication. Here, we use the novel, persistent keyword framework to identify instances where the keywords enter evolutionary medicine multiple times in the same year. When we compared the total publications associated with the multiple, independent, simultaneous innovations from 2007 through 2011 to all the publications from the same years, the innovative publications are significantly more likely to accumulate more citations per year than the total using an independent, unequal variance, single-tailed T-test ($p=0.006$). We also combined the innovative and similar publications to compare their citations to evolutionary medicine during those years, and we found, using the same statistical test, that with the innovative and similar publications combined, in an attempt to understand the larger knowledge landscape pertaining to these cases of simultaneous, independent innovations, are, again, significantly more likely to have more citations per year than the average evolutionary medicine publication ($p=8.104*10^{-6}$). This supports the findings in (Painter 2019) that the novel persistent keyword framework is an appropriate method for identifying innovations by indicating that the innovative papers are generating more attention than the average evolutionary medicine publication. Furthermore, Table 2 shows that except for 2007, the average citations for the innovative publications are consistently higher than the average citation counts for that year. 2007 may be lower due to the general nature and variety of metabolite research, and how it applies to evolutionary medicine. 2007 and 2010 also measure lower in average innovative citations than the average citation count for the similar publications. The high number of average similar citation counts indicates the topical knowledge landscape where metabolites and epigenome were introduced were popular and active areas of research.

**Table 3:** Innovative Keywords. 2008, 2010, and 2011 are instances of multiple independent, simultaneous innovations arising from shared collective knowledge pools

Year	Keyword	# of Papers	# of independent innovations	# of Distinct knowledge pools
2007	Metabolites	5	5	5
2008	Chromatin	3	3	2
2009	Triglyceride	5	5	5
2010	Epigenome	6	3	1
2011	Exome	3	3	2

From 2007 until 2011, we identified five instances of simultaneous, independent innovations. The following sections reveal through closer examination how the number of simultaneous, independent innovation events were determined. Table 3 lists the year and its keyword, the number of papers introducing the keyword, the number of independent innovation events, and the number of common collective knowledge pool. In order for a publication to represent an independent innovation event–as defined by introducing a novel keyword that persists in the corpus–the publication must not share any co-authors with other publications from a given year that also introduce the same keyword. We only consider co-authors for the particular year ($t = 0$) because previous collaborations would only contribute to the common collective knowledge pool *vis a vis* the diffusion of knowledge between co-authors, $K_{i1} \leftrightarrow K_{i2}$, and possibly between publication and domain, $K_p \longmapsto K_d$. The shared knowledge pool also includes shared references in the bibliography. Previous collaborations and shared references serve only to approximate the $K_i$ and $K_d$, respectively. Publications coming from a shared knowledge pool are still considered separate innovation events under the assumption is that if the co-author from past collaborations ($t < 0$) were a factor, then we should expect their participation at the time of the innovation ($t = 0$). Therefore, we use the common knowledge pool to simply provide an approximate measurement of the scientific zeitgeist surrounding the innovation in question.

### Metabolites

**Fig. 3:**
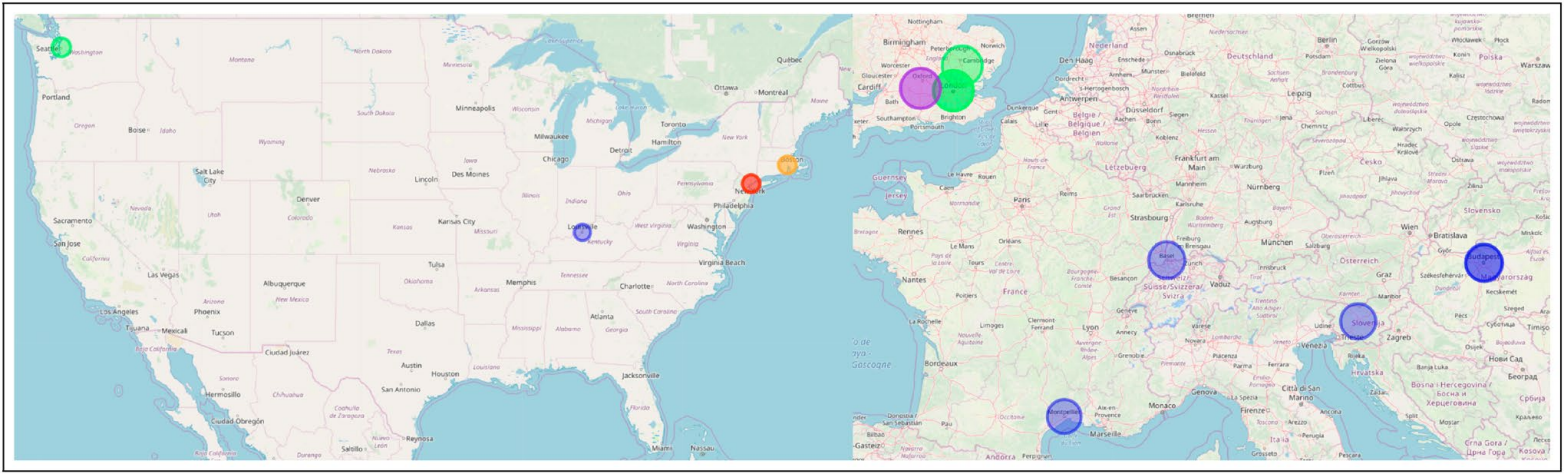
Location of Innovative Publications in 2007. Separate publications are denoted by color

Metabolites are small, intermediate molecules involved with metabolism. In 2007, the keyword metabolites was extracted for the first time, and subsequently every year after that. Of the five publications from which metabolites was extracted (Kőhalmy et al. 2007; Munkacsi et al. 2007; Min et al. 2007; McElwee et al. 2007; Jiang et al. 2007), we found no overlap in authors or references. The five papers did not cite each other. For our first case, we classify metabolites research entering evolutionary medicine as five independent, simultaneous innovation events. Because there was no overlap between the bibliographies, we conclude the innovative entrances of metabolites into evolutionary medicine originated from separate collective knowledge pools. We noted that (Min et al. 2007) and (Jiang et al. 2007) share an interest in longevity, and George C. Williams, a co-founder of evolutionary medicine, is most famous for his work on the evolution of senescence. (Williams 1957) The topic of aging has been identified by prominent scholars in evolutionary medicine as an active area of research. (Williams and Nesse 1991; Nesse 2001; Stearns 2005; Nesse 2008, 2011) Fig. 3 illustrates that author affiliations cluster spatially with concentrations in Europe (Kőhalmy et al. 2007), Great Britain (McElwee et al. 2007; Jiang et al. 2007), and the northeast region of the USA (Min et al. 2007; Munkacsi et al. 2007).

### Chromatin

**Fig. 4:**
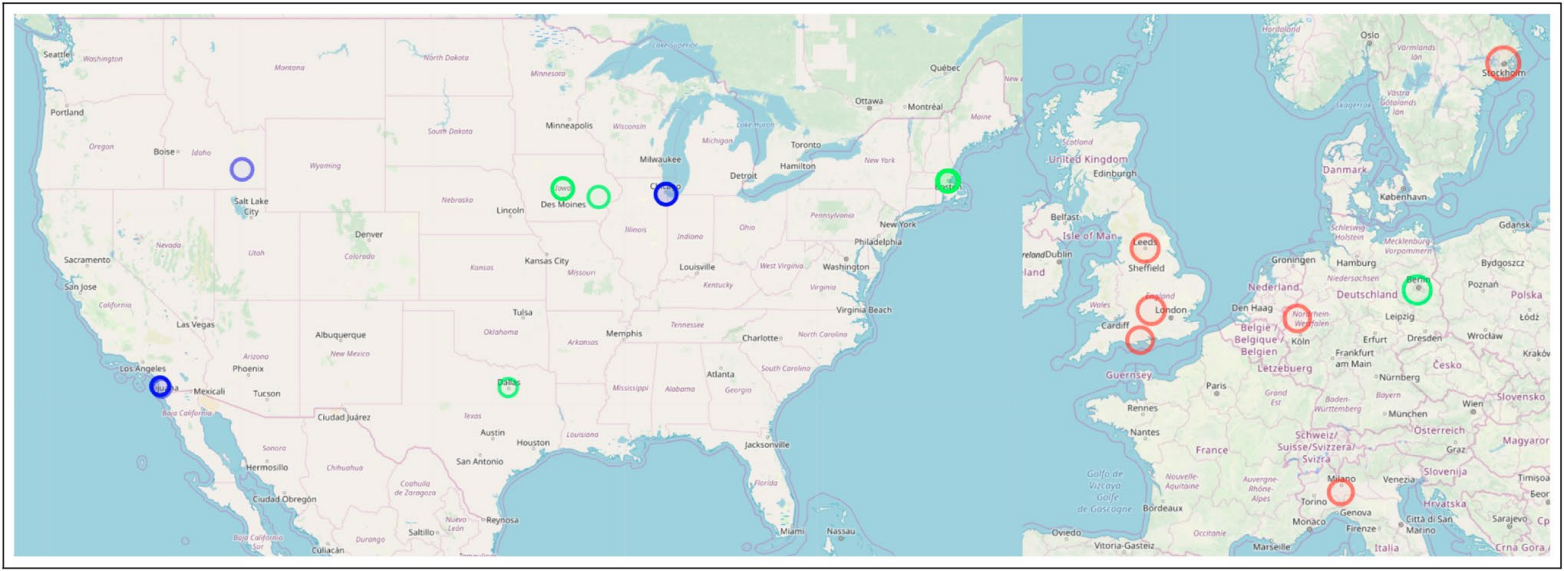
Location of Innovative Publications in 2008. Separate publications are denoted by color. Created using (Guide and Geocoding 2018)

Chromatin is condensed DNA found in eukaryotes that contains DNA, RNA, and proteins. 2008 saw chromatin extracted from three publications (Maeder et al. 2008; Zhou et al. 2008; Boultwood et al. 2008) for the first time and persist through the following years. There are no overlapping authors. Two publications (Zhou et al. 2008; Maeder et al. 2008) share the same references. Because they share references, we classify these together as having originated from the same common collective knowledge pool. Upon closer examination, both (Maeder et al. 2008) and (Zhou et al. 2008) use *drosophila melanogaster* as their model organism indicating a cursory level of shared collective knowledge. Therefore, the innovation of chromatin entering evolutionary medicine in three independent, simultaneous events arises from two separate common collective knowledge pools. Figure 4 indicates that two of the publications are affiliated in the USA (Zhou et al. 2008; Maeder et al. 2008), while the third is localized in Europe (Boultwood et al. 2008). A division that corroborates our assessment of two common knowledge pools with three innovations as specialized knowledge, like that needed to perform research in evolutionary medicine on chromatin, is known to cluster geographically (Feldman 1993; Martin and Moodysson 2011; van der and Rigby 2019). However, fruit flies are a staple model organism for geneticists.

### Triglyceride

**Fig. 5:**
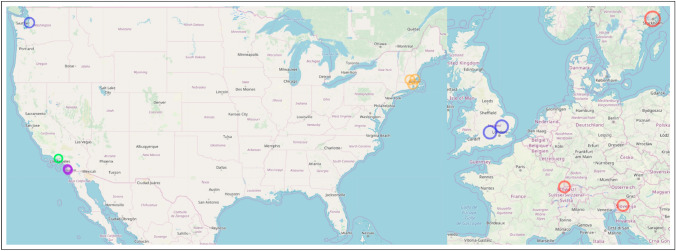
Location of Innovative Publications in 2009. Separate publications are denoted by color. Created using (Guide and Geocoding 2018)

Triglycerides are the main component of natural fats and oils. Similar to metabolites, triglyceride is also extracted from five different publications in 2009 and is extracted from at least one publication every subsequent year. (Schwimmer et al. 2009; Wang et al. 2009; Emerging et al. 2009; Režen et al. 2009; DiBello et al. 2009) Of the five publications in question, there is no overlap in co-authors or references, and they do not cite each other. Therefore, using our classification system, triglyceride entering evolutionary medicine is five independent, simultaneous innovation events that share no common collective knowledge pool. Separate knowledge pools may indicate that they were introduced into evolutionary medicine for different purposes to understand different problems. (Schwimmer et al. 2009; Emerging et al. 2009), and (Režen et al. 2009) are all research articles pertaining to fats and liver health. No shared references between the three indicate a variety of research agendas on the subject. (Wang et al. 2009) is a study on rat lung maturation, and (DiBello et al. 2009) are interested in metabolic syndrome in Samoans. A major issue in evolutionary medicine is the mismatch between how our metabolisms evolved and our increasingly sedentary lifestyle. (Nesse 2011; Rühli et al. 2016; Gluckman et al. 2016) All five of these publications help to illuminate the complex role of triglycerides within evolutionary medicine. Again, we observe in Fig. 5 that the affiliations from these publications cluster together in Europe (Režen et al. 2009), Great Britain (Emerging et al. 2009), and on the east (Wang et al. 2009; Schwimmer et al. 2009) and west coasts (DiBello et al. 2009) of the USA .

### Epigenome

**Fig. 6:**
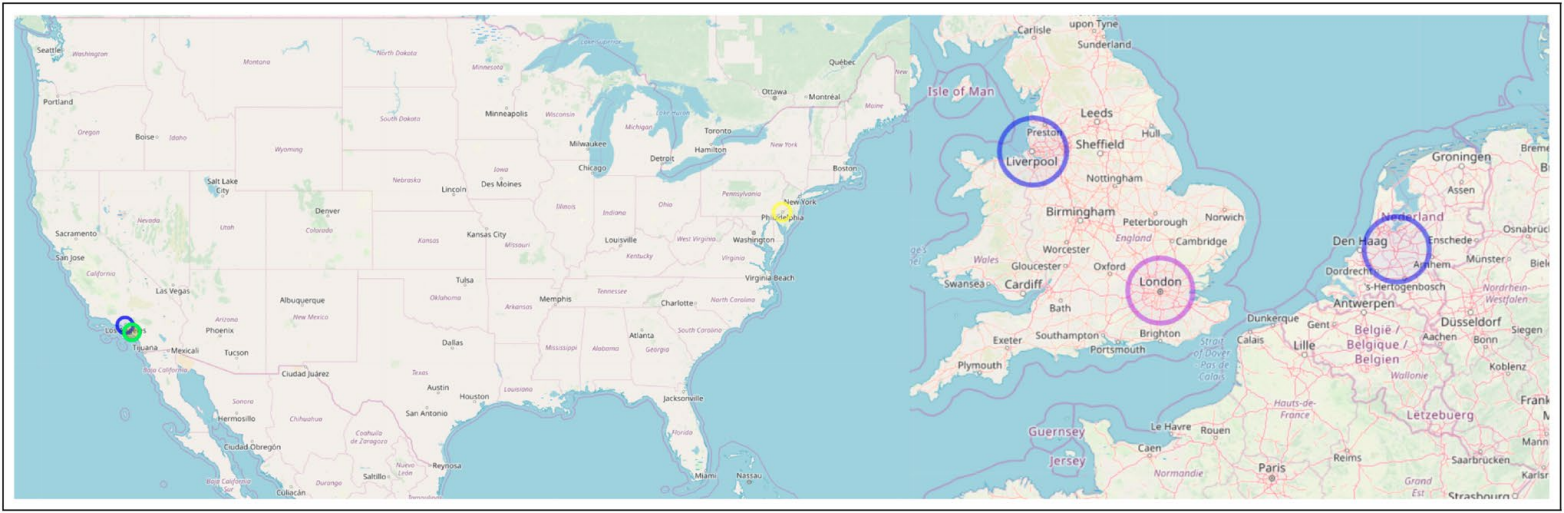
Location of Innovative Publications in 2010. Separate publications are denoted by color. Created using (Guide and Geocoding 2018)

In 2010, epigenome was extracted as a novel persistent keyword from six publications (De Magalhães et al. 2010; Wallace 2010a, b; Wallace et al. 2010; Wallace and Fan 2010; Bell and Beck 2010). Of those six publications, four shared co-authors (Wallace 2010a, b; Wallace et al. 2010; Wallace and Fan 2010) and three (Wallace 2010a, b; Wallace et al. 2010) shared citations between each other. Therefore, (Wallace and Fan 2010) is the first of the four as the others were not yet published and not available to cite. This linear progression of citations clearly indicates the publications are not simultaneous but are, in fact, sequential. The shared authorship further supports this claim as many of the same authors appear on all four publications. All six of the innovative publications shared common references. Therefore, we grouped the four publications that shared authors and cited each other into a single event spearheaded by (Wallace 2010b) which was not cited by the other three, indicating it was published first. The remaining two publications only shared citations. This indicates there were three independent, simultaneous innovation events, (De Magalhães et al. 2010; Wallace and Fan 2010), and (Bell and Beck 2010), which originated from a single common collective knowledge pool. A single pool is indicative of simultaneous, independent innovations produced as a result of an intellectual ecosystem ripe for innovation. The epigenome consists of the regulatory control elements that up-regulate and down-regulate genes in gene regulatory networks. (Wallace 2010a, b; Wallace et al. 2010), and (Wallace and Fan 2010) (the four sharing authors and citations) are all focused on different aspects of mitochondrial genetics. (De Magalhães et al. 2010) use next-gen sequencing to study aging, and lastly, (Bell and Beck 2010) studied the link between the epigenome, the environment, and disease. Similar to the previous three cases, Fig. 6 shows regional clusters of affiliations based on paper in Europe (De Magalhães et al. 2010; Bell and Beck 2010) and the west coast of the USA (Wallace 2010a, b; Wallace et al. 2010; Wallace and Fan 2010).

### Exome

**Fig. 7:**
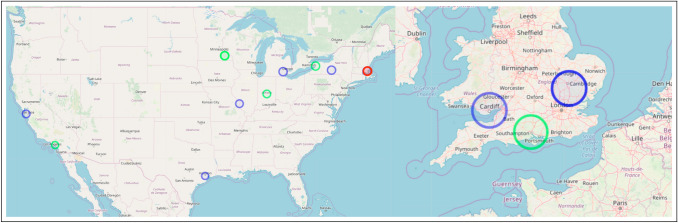
Location of Innovative Publications in 2011. Separate publications are denoted by color. Created using (Guide and Geocoding 2018)

The final novel, persistent keyword we identified was exome. The genome consists of two main parts, (a) the introns, which are removed before the RNA coding regions, and (b) the exons are processed. The exome consists of only the exons. Exome was extracted from three different publications that shared no co-authors. (Marth et al. 2011; Stranger et al. 2011; Klein et al. 2011) Two of the publications shared references (Marth et al. 2011; Stranger et al. 2011), and none of the publications in question cited each other. Consistent with our classification thus far, there were no shared co-authors and no cross-citations, so we classify exome as entering evolutionary medicine in three independent, simultaneous innovation events originating from two separate collective knowledge pools. Again, two collective knowledge pools for three independent innovations are likely the result of an intellectual zeitgeist producing a moment in time ready for innovation. (Marth et al. 2011) is a study on low-frequency genetic coding variation, and (Stranger et al. 2011) examines genome-wide association of complex genetic traits. The third publication, (Klein et al. 2011), is a medical trial on dementia with hearing loss. Two papers contained a variety of affiliations spread out across the USA and Europe (Klein et al. 2011; Marth et al. 2011), but the third is localized in the northeast of the USA (Stranger et al. 2011) (Fig. 7).

### Geographic concentration and dispersion

**Fig. 8:**
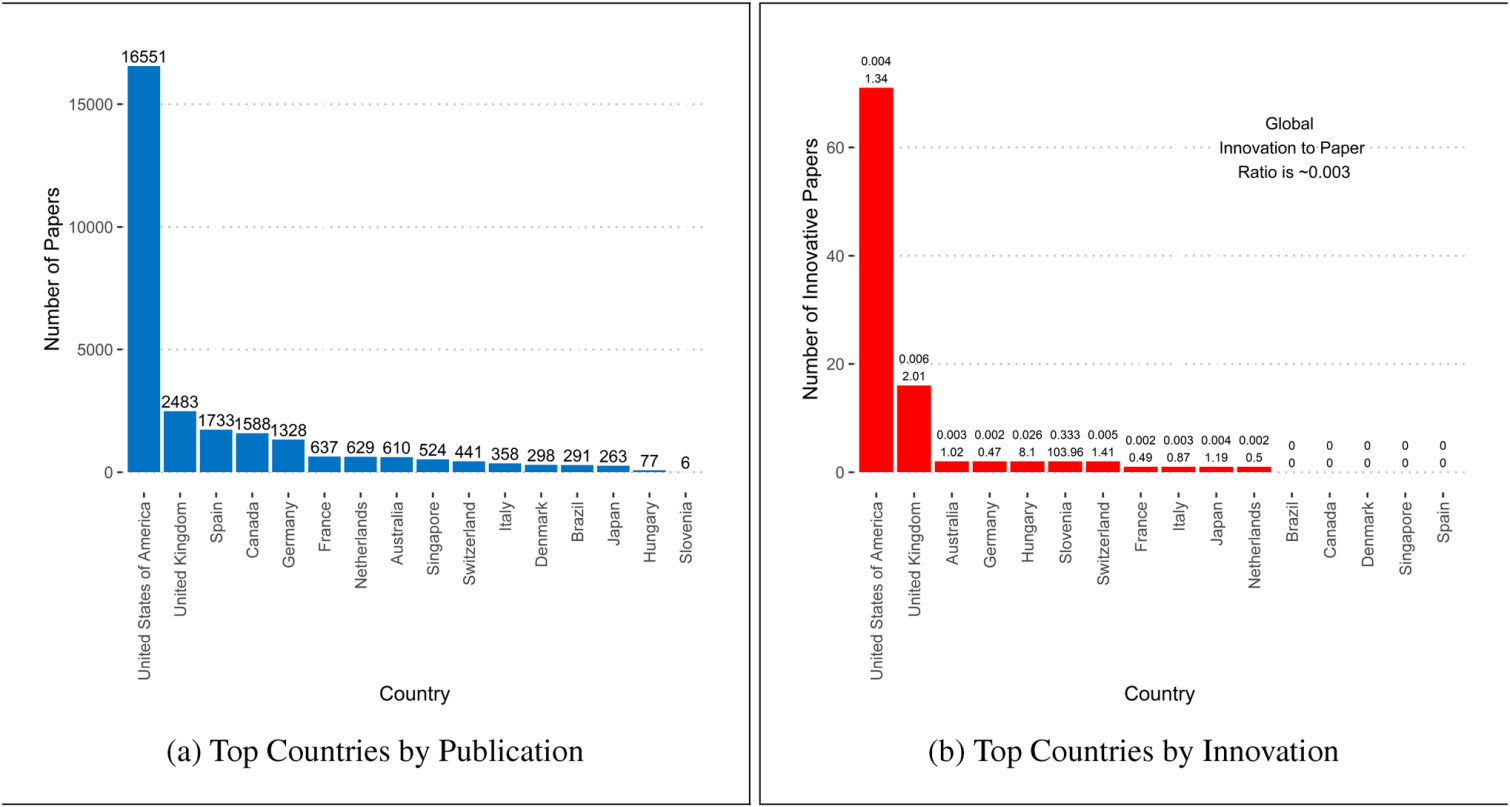
Author Affiliations of Evolutionary Medicine. The numbers above the bars in **a** indicate the number of publications. The top number above the bars in **b** is the ratio of innovative to non-innovative publications, and the bottom number is the innovative ratio compared to the global average

Similar to the human population (Seldin et al. 2006; Small and Nicholls 2003; Small and Cohen 2004), economic activity (Feser and Sweeney 2000; McCann and Van Oort 2019; Li et al. 2019) and technological innovation (Malmberg et al. 1996; Audretsch et al. 2004; Turkina and Van Assche 2018; van der Wouden and Rigby 2019) are academic publishing in the field of evolutionary medicine concentrated in space. Using the author affiliations provided in the Web of Science metadata, Fig. 8a shows that most papers in this field are produced in North America and Europe, with the USA leading the way. Australia, Singapore, Brazil, and Japan are the only countries in the Top 16 that are located on different continents. Note that this spatial distribution does not simply follow the distribution of human population across space. Countries with large population numbers such as China, India, and Pakistan produce only very small numbers of papers in this field. Instead, this distribution shows which countries have (academic) interests in evolutionary medicine and the capabilities and means to publish in the field.

The innovative papers in evolutionary medicine are even more geographically localized than the field in general. The global innovative-to-non-innovative papers ratio in evolutionary medicine between 2007 and 2011 was 0.003. This means that only roughly three out of 1000 papers in this field produces an innovation. Figure 8b indicates that the USA, followed by the UK and Australia, are producing the most papers identified as innovative. For each country the innovative-to-non-innovative paper ratio is shown (top) and compared to the global average (bottom). This later ratio indicates how “efficient” (ratio > 1) a country is in producing in innovation in evolutionary medicine, compared to the global average. While the top three countries producing the most innovative have ratios greater than 1, Slovenia and Hungary are exceptionally efficient. In Slovenia 33% of the papers published in evolutionary medicine is considered an innovation, 106 times the global average. In Hungary roughly 2.6% of the papers is an innovation - 8 times the global average. While these percentages are impressive, we must note the relatively few publications with affiliations to these countries.

The production of knowledge, including academic publication, is increasingly the outcome of collaboration (Wuchty et al. 2007; Schultz-Jones 2009; van der Wouden 2019). All our innovative papers in evolutionary medicine are co-authored papers. If authors of the innovative papers are truly independent and draw from a common collective knowledge pool, the geographical distribution of the co-authors should not reflect distinct spatial patterns. For instance, if the authors of the innovative papers all cluster in space while the authors of comparable non-innovative papers are dispersed across space, this is a signal that there might be specific localized knowledge. This goes against our claim of the common collective knowledge pool. By definition, geographically localized knowledge suggests an exclusion of certain knowledge from a concept-specific knowledge pool. Should we find that authors of innovative papers cluster more closely, this would suggest that their innovative contributions were not the result of an intellectual ecosystem produced by a common knowledge pool and, instead, the result of some geographically localized knowledge is only available to those in close proximity.

**Fig. 9:**
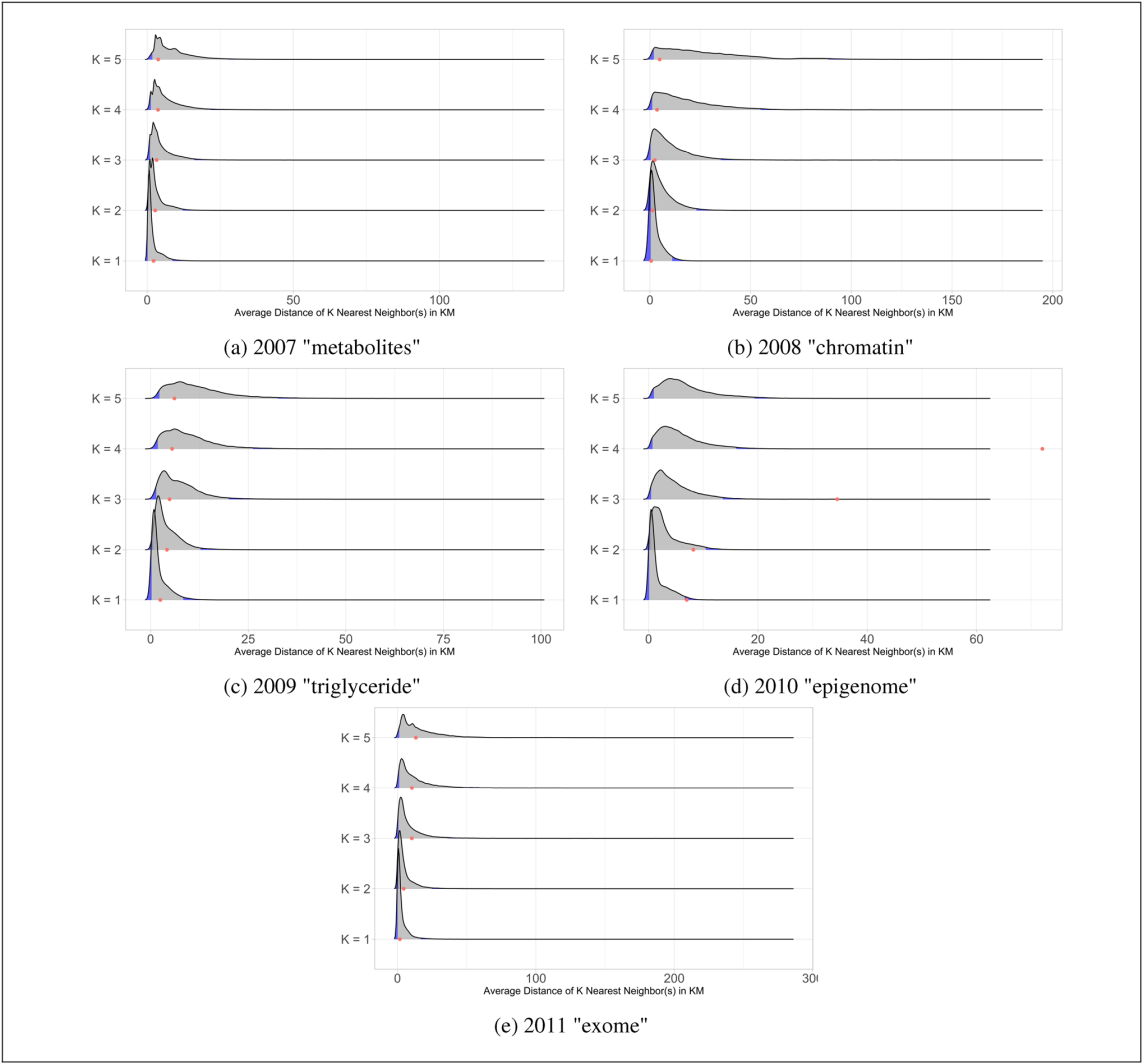
Geographical Distance of k-Nearest Neighbors for Innovative Papers (Red) with Bootstrapped Distribution for Non-Innovative Papers on same Topic. The distribution of distances is shown in gray for the innovative publications and their similar counterparts that share non-innovative keywords in common. The innovative publications are shown with red dots. The position of the dots indicates no significant difference between the geographical distances of co-authors of innovative and non-innovative publications

To examine whether innovative papers are more likely to cluster in space than similar non-innovative papers, we calculate the average geographical distance between the five nearest co-authors on each paper. The red dots in Fig. 9 plot the average geographical distance to the first five nearest co-authors on innovative papers. The gray distributions reflect the average geographical distance to the k-nearest co-authors for authors on non-innovative papers on the same topic bootstrapped 50,000 times. The blue shaded parts of the distribution reflect the bottom 2.5% and top 97.5% of the distribution. When the red dots are located in this area, it means that the co-authors of innovative papers are significant more likely to be clustered (bottom 2.5%) or dispersed (top 97.5%) than compared to similar non-innovative papers. We find no significant spatial clustering of authors on innovative papers. Therefore, we do not find evidence that these innovative publications are the product of certain unique localized knowledge. This is supported by previous research, suggesting the advent of modern technology has decreased the role geographical distance in collaborations in favor of position within cognitive and social networks (Boschma 2005; Ooms et al. 2018; van der Wouden and Rigby 2019). However, this finding contradicts recent evidence indicating that geographical proximity promotes flows of complex, tacit types of knowledge that are often seen as the key inputs for innovation (Balland et al. 2015; van der Wouden and Rigby 2019).

## Discussion

Identifying and classifying simultaneous, independent innovations goes far beyond simply making sure researchers receive proper credit for their research. Quantifying this particular kind of innovation strengthens our knowledge and understand of how scientific innovations are created and how they behave once they are released into the world. This begins by applying a strict operational definition on what it means to be a simultaneous, independent innovation. Here, we adapted the novel, persistent keyword framework previously used to identify and rank innovations (Painter 2019) to identify simultaneous innovations. After they were identified, we classified them as independent and characterized the foundational knowledge–a common collective knowledge pool–for each innovation event. We identified approximately 100 similar publications for each set of independent, simultaneous innovation events based on the number of non-innovative keywords were shared with the innovative publications. Furthermore, we identified where these authors are affiliated and mapped the clustering of individual knowledge geographically.

To be considered an innovation under the novel, persistent keyword framework, when a new keyword appeared in the corpus, it must appear in every year following its introduction–novelty and persistence. This follows Schumpeter and Brozen characterization of invention, innovation, and imitation. (Brozen 1951; Shumpeter 1942) Once more, the innovation is not the invention of a new keyword. The innovation is the novelty, incorporation, and adoption of keywords presumably from other scientific domains not previously present in the corpus. The innovations are considered independent if the publications do not share the same co-authors, and simultaneous if they appear in the same year and do not directly cite each other. A citation in this matter would indicate that the publication containing the reference to the other was published after ($t+1$), implying it was not the original point of entry into evolutionary medicine.

If two publications share an author, that individual acts as a kind of knowledge bridge linking the two groups of authors in time and space. Therefore, we group these kinds of publications into a single innovation event. However, should the authors of the publications introducing the novel, persistent keyword share authors in the past, they are not grouped into a single event and instead are still considered two separate events. The publications would be considered to have arisen from a common collective knowledge pool partially influenced by the sharing co-author in the past. We justify this with the assumption that previous collaborations do not necessarily dictate future work. It is reasonable to assume two individuals collaborate in the past, and later they independently reach the same solutions to a given problem.

The concept of the common collective knowledge pool is of particular importance if one were one to adopt the viewpoint that some innovations are a result of the knowledge landscape in which they are situated. (Galton 1874; Merton 1973; Simonton 2010) Navigating this innovation landscape is an area of active research. (Chesbrough 2006; Sandstrom and Bjork 2010; Huang 2010; Bogers et al. 2017; Curley and Salmelin 2018; Frishammar et al. 2019) The common collective knowledge pool is a representative proxy of this knowledge landscape. In this study, chromatin, epigenome, and exome enter evolutionary medicine from their respective shared common collective knowledge pools. This can be interpreted as the knowledge landscape between evolutionary medicine each of the respective keywords was such that it made their introduction more likely. The scientific zeitgeist of evolutionary medicine was fertile and waiting for these keywords to be incorporated into the field setting into motion their subsequent adoption in the future, while also signaling an innovation had occurred.

Following this train of thought, simultaneous, independent innovations imply a sort of void in the knowledge landscape–a vacant conceptual niche–that is filled by research involving these keywords. Because these keywords are first introduced multiple times in the same year independently, it is reasonable to conclude there is something about these keywords, and the concepts they represent, that was seen by multiple researchers as being directly relevant to evolutionary medicine at the same time. Likely without deliberate intention to introduce new keywords into evolutionary medicine, the authors of the 22 papers we examined filled an empty conceptual niche that was, presumably inadvertently, created by themselves and their evolutionary medicine peers.

Let us shift from intellectual niches to an analogy about convergent and parallel evolution in species. While both indicate that two species independently arrived at similar evolutionary traits, convergent evolution supposes that distantly related species are likely to have different underlying genetics than closely related species, as is the case in parallel evolution. The name for a trait that evolves by convergent or parallel evolution is homoplasy or analogous structure. Biologists will quickly point out that it is likely parallel and convergent evolution happen in response to similar selective pressures. (Arendt and Reznick 2008; Elmer and Meyer 2011; Pearce 2011; Stern 2013; Foote et al. 2015; Bailey et al. 2015)

There is also evidence for convergent and parallel cultural evolution. Humans domesticated similar crops on separate continents. (Fuller 2014) Similar words exist in languages completely isolated from one another. (Dingemanse et al. 2013) In addition, there is evidence that several cultures invented bladed tools independently. (Jennings and Smallwood 2018) These are examples of different cultures, faced with similar problems, that all found similar solutions.

We quantified multiple, simultaneous, independent innovations. The creation of those innovations is similar to the convergent and parallel evolution of an adaptation. Convergent evolution implies two distant species evolving the same trait. This is similar to two separate publications introducing the same keyword with separate publication knowledge pools ($K_p$), as we observed in 2007 and 2009. Alternatively, parallel evolution exists when two closely related species independently evolve the same trait. Following the analogy, parallel evolution is similar to what we observe in 2008, 2010, and 2011 (see Table 3), when several publications were measured as having the same common collective knowledge pool independent and simultaneously introduced the same novel keyword that will go on to persist in the evolutionary medicine corpus.

This brings the analogy to its conclusion. Homoplasies in biology and human society evolve due to similar selective pressures from being faced with similar problems. Our evidence supports that the keyword homoplasies in the evolutionary medicine corpus occurred due to the selective pressures imposed on the researchers by the intellectual ecosystem of evolutionary medicine as evidenced by the five instances of simultaneous independent innovations described here.

We wish to conclude our discussion of these findings with a reflection on the variable nature of citation and collaboration practices across scientific disciplines and the interdisciplinary nature of evolutionary medicine. It is well reported in multiple studies that citation and collaboration practices can vary significantly between scientific disciplines (Barabâsi et al. 2002; Newman 2001, 2001a, b; Elliott 1981; Small 1999; Kousha and Thelwall 2007; Hyland and Jiang 2019; Chen 2017). This, in concert with empirical and anecdotal evidence of the interdisciplinary nature of evolutionary medicine (Painter 2019; Painter et al. 2021), requires that we include the caveat that this is research is but one study about one scientific discipline over a limited time span. While tempting to draw generalizations it may be, more research with other data sets and time frames is required and indeed in progress.

## Conclusion

By classifying an innovation as a publication that introduces a new keyword that the keyword then persists thereafter, we demonstrate how that framework can be leveraged to identify simultaneous, independent innovations. We classify innovations as simultaneous if they are published in the same year. Furthermore, we support independence through rigorous geographical comparisons and metadata analysis. We map the location of the authors responsible for introducing the same novel, persistent keywords in the same year. We found that while specialized knowledge may cluster in certain areas, there was no significant clustering between innovative authors. Persistent novelty is only one particular type of innovation on a spectrum. We find instances of simultaneous, independent innovations arising from collective knowledge and independent knowledge pools emphasizing the importance of independent thought as well as attention to the scientific culture in which they are embedded. In conclusion, the understanding the conceptual landscape can only illuminate the door to the innovation, someone must still walk through it. Nevertheless, it may turn out that promoting innovation is less about finding someone to stand on the shoulders of giants and more about finding where the giants are standing.
